# Experimental evidence for a semantic typology of emoji: Inferences of co-, pro-, and post-text emoji

**DOI:** 10.1177/17470218241255786

**Published:** 2024-08-19

**Authors:** Lyn Tieu, Jimmy L Qiu, Vaishnavy Puvipalan, Robert Pasternak

**Affiliations:** 1 Department of French, University of Toronto, Toronto, Ontario, Canada; 2 MARCS Institute for Brain, Behaviour and Development, Western Sydney University, Penrith, New South Wales, Australia; 3 Department of Linguistics, Macquarie University, Sydney, New South Wales, Australia; 4 Teachers College, Columbia University, New York, NY, USA; 5 Deepset, Berlin, Germany

**Keywords:** Emoji, gesture, projection, semantics, pragmatics, inferences, presupposition, supplements

## Abstract

Emoji symbols are widely used in online communication, particularly in instant messaging and on social media platforms. Existing research draws comparisons between the functions of emoji and those of gestures, with recent work extending a proposed typology of gestures to emoji, arguing that different emoji types can be distinguished by their placement within the modified text and by their semantic contribution (the linguistic inferences that they give rise to). In this paper, we present four experiments designed to test the predictions of this extended typology, the results of which suggest that emoji symbols indeed trigger the hypothesised linguistic inferences. The findings provide support for a semantic typology of emoji and contribute further evidence of the parallels between gesture and emoji.

## Introduction

Emoji are graphic symbols that are widely used in online communication, including instant messages on various platforms, in emails, text messaging, and in social media posts (Dresner & Herring, 2010). Emoji can represent a wide diversity of concepts and objects, corresponding to facial expressions, animals, plants, activities, gestures and body parts, as well as emotions and feelings, and even more abstract concepts (Rodrigues et al., 2017). The first set of emoji was created by Shigetaka Kurita, and released in 1999 (Bai et al., 2019). The word *emoji* is a transliteration of the Japanese word 絵 (*e* = picture) 文字 (*moji* = character).

Over half of all online messages on platforms like Instagram reportedly contain emoji (Dimson, 2015), and this figure has almost certainly increased in the last decade. In terms of their communicative functions, a review by Bai et al. (2019) reports that emoji can serve multiple different functions, including the establishment of emotional tone, the reduction of discourse ambiguity, the enhancement of context appropriateness (Kaye et al., 2016), and the intensification or weakening of speech acts (Sampietro, 2019).

In this study, we focus on the linguistic contributions of emoji symbols that denote objects and activities, and in particular on their semantics. Our study asks what such emoji contribute to the meanings of the sentences that they modify. The starting point for the study is an observation by Pierini (2021) that emoji symbols denoting objects (e.g., the balloon emoji 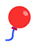) and activities (e.g., the dancing emoji 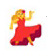) interact with logical operators, such as disjunction, conjunction, and negation, when co-occurring with text. Such emoji appear to interact with logical structure in a way that looks to be comparable to how manual gestures reportedly interact with speech. Pierini proposes a semantic typology of emoji that is based on a typology of gestures previously proposed by Schlenker (2018a, 2018b).

Schlenker’s typology includes *co-speech* gestures, *pro-speech* gestures, and *post-speech* gestures, exemplified in (1)–(3). In each example, the gesture is indicated in all-caps font and is aligned differently with the modified speech: in (1), it is produced simultaneously with the words “worked out”; in (2) it fully replaces the words “worked out”; and in (3) it occurs after the spoken words “worked out”. In all three cases, the gesture seems to contribute more detailed information about the nature of Paige’s workout, something like *lifting weights*, as in (4).

(1) Today, Paige [worked out]_ (Co-speech gesture)
   LIFT-WEIGHT
(2) Today Paige LIFT-WEIGHT (Pro-speech gesture)
(3) Today, Paige worked out— (Post-speech gesture)
   LIFT-WEIGHT
(4) ⤳ *Paige’s workout involved lifting weights*

Schlenker (2018b) proposes to distinguish the three types of gestures by two criteria. The first is whether the gesture is *external* or *internal*, that is, whether its contribution is semantically and syntactically optional. While co-speech and post-speech gestures are considered *external* (they can be ignored or removed without altering the completeness and acceptability of the modified sentences), *pro-speech* gestures are considered *internal*, since their removal would result in a syntactically and semantically incomplete sentence.

The second property that distinguishes the gesture types is whether they occupy an independent time slot. While co-speech gestures do not, since they occur simultaneously with spoken words, pro-speech and post-speech gestures do occupy their own independent time slot, as they either fully replace a word or phrase (pro-speech) or follow spoken words (post-speech).

In basic affirmative sentences like (1)–(3), all three gestures, regardless of their alignment in the sentence, seem to make the same semantic contribution—they contribute an inference about the nature or activity of Paige’s workout. What Schlenker observes, however, is that once we add logical operators to the modified sentences, such as negation (“not” or the suffix “-n’t”), modals (e.g., “might”), and quantifiers (e.g., “each”, “none”, and “exactly one”), each of the three gesture types interacts in particular ways with these logical operators. By studying their interactions, we learn that each gesture type makes a different kind of semantic contribution.^1^ According to Schlenker, (i) co-speech gestures contribute “cosuppositions”, or *conditionalised assertion-dependent presuppositions*, (ii) pro-speech gestures contribute at-issue content and can trigger standard presuppositions, and (iii) post-speech gestures contribute what are known as *supplements*. Let us consider each in turn before moving on to an extension of the typology to emoji.

First, co-speech gestures are produced simultaneously with speech, and trigger cosuppositions, which crucially project from embedded environments in much the same way that standard presuppositions do. Take the standard presupposition of the factive attitude verb “regret”, as in (5). If we embed the trigger under negation, as in (5b), the presupposition that *X quit* projects, such that the negative statement still presupposes that *X quit*. That is, the presupposition does not seem to be affected by the negation, leading to its well-known behaviour of *projection*. There is also experimental evidence that in the scope of quantifiers like “none”, presuppositions project *universally* (Chemla, 2009); that is, (5c) presupposes that *each* of the relevant students quit.

(5) Standard presuppositions
  a. X regrets quitting.
   Presupposes: *x quit*
  b. X doesn’t regret quitting.
   Presupposes: *x quit*
  c. None of my students regrets quitting.
   Presupposes: *Each of my students quit*

Schlenker (2018a, 2018b) observes that co-speech gestures trigger something very similar to presuppositions. The sentence (6a), with the UP gesture co-occurring with the words “uses the stairs” triggers the inference that if x uses the stairs, x goes up. And according to Schlenker, this cosupposition also projects, like presuppositions do. Thus, the negative (6b) with the UP gesture co-occurring with “use the stairs” still presupposes that if x were to use the stairs, x would go up. Like standard presuppositions, cosuppositions also project from the scope of quantifiers. So the quantified (6c) presupposes that for all of the students, if they use the stairs, it is to go up.

(6) Cosuppositions (conditionalised presuppositions)
  a. x [uses the stairs]_ 
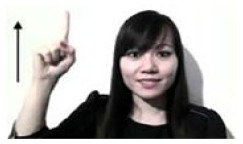
   ⤳ *If x uses the stairs, x goes up*
  b. x doesn’t [use the stairs]_ 
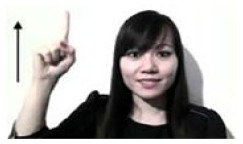
   ⤳ *If x uses the stairs, x goes up*
  c. None of my students [uses the stairs]_
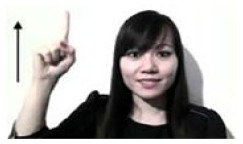
   ⤳ *For each of my students, if they use the stairs, they go up*

Schlenker (2018a, 2018b) formalises these intuitions within a dynamic semantics (Heim, 1983; Schlenker, 2009), according to which presuppositions must be satisfied in their local contexts. In basic terms, co-speech gestures trigger presuppositions that their content is entailed by that of the expressions that they co-occur with. These presuppositions, however, are *conditionalised* on the assertive content of the modified expressions.

Adopting a presuppositional view of co-speech gestures predicts that the cosuppositional inferences they trigger should project from linguistic environments from which standard verbal presuppositions typically project, and this prediction has been investigated and supported by experimental studies. Tieu, Pasternak, Schlenker, and Chemla (2017, 2018) present experimental evidence from truth value judgement, picture selection, and inferential judgement tasks showing that co-speech gestures trigger cosuppositional inferences, which importantly project from embedded environments including the scope of negation, the modal “might”, and the quantifiers “each”, “none”, and “exactly one”.

Next, consider pro-speech gestures, which fully replace spoken words. Schlenker (2018b) observes that pro-speech gestures, particularly those that indicate the shape of an object, trigger standard presuppositions (for experimental evidence of this, see Tieu et al., 2019). For example, the sentence in (7), in which the words “turn the wheel” are replaced by the gesture of turning a wheel (represented as TURN-WHEEL below), seems to trigger the presuppositional inference that John is currently behind the wheel. Importantly, this inference appears to project from environments from which standard presuppositions typically project, such as negation (8) and questions (9), and moreover, projects universally from the scope of the quantifier “none” (10), as standard presuppositions are argued to project (Chemla, 2009).

(7)  John is going to TURN-WHEEL
  ⤳ *John is currently behind a wheel*

(8)  John is not going to TURN-WHEEL
  ⤳ *John is currently behind a wheel*

(9)  Is John going to TURN-WHEEL?
  ⤳ *John is currently behind a wheel*

(10) None of John’s friends is going to TURN-WHEEL
  ⤳ *Each of John’s friends is currently behind a wheel*

Finally, post-speech gestures, which fully follow the speech that they modify, are argued by Schlenker to display the behaviour of appositive relative clauses, thus triggering *supplements*. Supplements are inferences that must make non-trivial contributions (Potts, 2005) and differ from entailments, implicatures, and presuppositions in that they seem not to interact much with logical operators (Schlenker, 2018a, 2018b).

Schlenker (2019) provides the example in (11), involving a slapping gesture following the sentence. The gesture contributes a supplemental inference about the slapping nature of the punishment, and like standard supplements, this inference projects from the antecedents of conditionals, as seen in (12).^2^

(11) Mary will punish her enemy – 
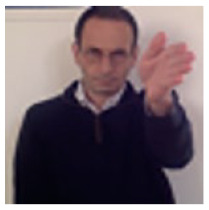
(12) If Mary punishes her enemy – 
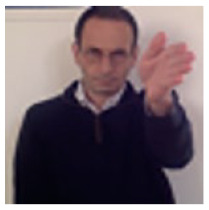
,we’ll hear about it.
  ⤳ *If Mary punishes her enemy, slapping will be involved*.

In sum, Schlenker (2018b) provides a typology of three types of gestures, which can be distinguished by whether their contribution is semantically and syntactically optional and whether they occupy an independent time slot. It is moreover observed that the three types of gestures interact in different ways with logical operators in the sentences that they modify, giving rise to different semantic contributions. The three types of gestures can be summarised in Table 1.

**Table 1.** Schlenker’s (2018) typology of gestures.

Gesture type	External/internal	Independent time slot	Semantic contribution
Co-speech	External	No	‘Cosuppositions’ (conditionalised assertion-dependent presuppositions)
Pro-speech	Internal	Yes	At-issue, can trigger standard presuppositions
Post-speech	External	Yes	Supplements

## A typology of emoji

In parallel with gestures, Pierini (2021) observes that emoji that denote objects and activities can similarly pattern into three types, likewise distinguishable by their alignment with the modified text and their semantic contribution. Importantly, he observes that emoji can interact with logical operators like negation and quantifiers in very similar ways to gestures. We will refer to Pierini’s (2021) typology as the “Extended Typology”.

Within the Extended Typology, co-text emoji are the textual equivalent of co-speech gestures; they should thus co-occur with the relevant modified text. Due to the linearisation constraints of written text, however, emoji cannot technically occur simultaneously with written text. As pointed out by Gawne and McCulloch (2019) and Cohn et al. (2019), gestures (and co-speech gestures, in particular) display a tight temporal synchrony with speech (Church et al., 2014; Habets et al., 2011; Kelly, 2014), and this same temporal relationship is impossible in the written domain. Co-text emoji, however, can appear after the relevant text, as in (13a), or they can “bracket” the modified text as in (13b).^3^ In both cases, the inference that is triggered is that the match was a soccer match (13c).^4^

(13) a. Canada lost the match 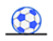  b. Canada 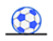 lost the match 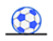  c. ⤳ *Canada lost the soccer match*

In terms of the semantic contribution of co-text emoji, like co-speech gestures, these are argued to trigger cosuppositional inferences. These inferences are observed to project from embedded environments, such as the scope of modals, questions, negation, and quantifiers, just like standard presuppositions. Thus, the LIFT-WEIGHT emoji 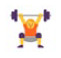 in (14a) provides information about Paige’s workout, and this inference about weight-lifting persists in the embedded environments in (14b) through (14e).

(14) Examples of cosuppositional inferences
  a. Paige worked out today 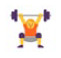     ⤳ *If Paige worked out today, it involved weightlifting*

b. Modal: Paige might work out today 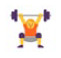   ⤳ *If Paige works out today, it will involve weightlifting*

c. Question: Did Paige work out today? 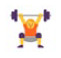   ⤳ *If Paige worked out today, it involved weightlifting*

d. Negation: Paige did not work out today 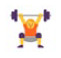   ⤳ *If Paige had worked out today, it would have involved weightlifting*

e. Quantifier: None of the students worked out today 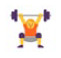   ⤳ *For each of the students, if they had worked out, it would have involved weightlifting*

Next, consider pro-text emoji, which are the equivalent of pro-speech gestures within the typology. Similarly to how pro-speech gestures can fully replace spoken words, emoji symbols can fully replace a word or phrase, as in the examples Pierini (2021) provides from Twitter:

(15) She is the 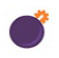  ⤳ *She is the bomb*

(16) Sleepy and tired. . . all I want is my 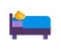  ⤳ *Sleepy and tired . . . all I want is my bed*

Just as pro-speech gestures are argued by Schlenker to contribute an at-issue semantics, according to Pierini, pro-text emoji likewise contribute an at-issue semantics. Pro-text emoji essentially contribute the kinds of meanings that normal written words would.^5^ The meaning of (17) is essentially the same as if the words “lifted weights” had been produced in place of the emoji.

(17) Yesterday, John 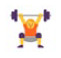 for two hours.
  ⤳ *Yesterday, John lifted weights for two hours*

Like normal words, some pro-text emoji will trigger presuppositions. Pierini provides the example in (18), in which the SLEEP emoji 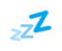 contributes to the meaning of *sleep*, but also triggers the presupposition that Mary is currently awake.

(18) In two minutes, Mary will soon 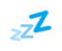  ⤳ *Mary is currently awake*

**Figure 1.**
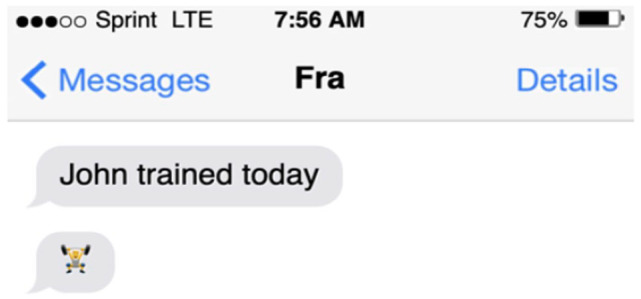
Screen capture of Pierini’s example of post-text emoji (Pierini, 2021, p. 727).

Finally, just as post-speech gestures follow the speech they modify, post-text emoji fully follow the written text that they modify. As Pierini (2021) observes, the emoji and the modified text can be separated by an ellipsis or a dash, as in (19) and (20), or, in a more naturalistic text messaging sequence, the emoji could appear after the relevant text by popping up in a subsequent text message, as in Figure 1.

(19) John trained today. . . 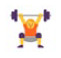  ⤳ *John trained today, which by the way involved weightlifting*

(20) John trained today – 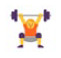  ⤳ *John trained today, which by the way involved weightlifting*

In all three cases, the inference generated is that John’s training today involved weightlifting.

In terms of their semantic contribution, post-text emoji are argued to contribute supplements, just like post-speech gestures do.^6^ Supplements are known to be degraded in negative environments (see Potts, 2005 for one view as to why, which relates to the observation that supplements cannot interact scopally with logical operators). For our purposes, it suffices to note that the negative version of the post-text emoji sentence in (19), as in (21), is expected to be unacceptable, and according to Pierini (and our intuitions), it is indeed a bit odd.

(21) # John didn’t train today. . . 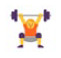

Finally, as we have seen in the case of gestures, supplements are known to project from the antecedents of conditionals. The examples in (22a) and (23a) trigger supplemental inferences, which project from the antecedents of the conditionals as in (22b) and (23b).

(22) a. If the professor is interrupted during her lecture. . . 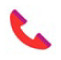, she will end the lecture early.
*b. *⤳ If the professor is interrupted during her lecture, it’ll be because of a ringing phone**

(23) a. If the businesswoman travels to the board meeting. . . 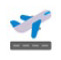, the company will reimburse her expenses.
b. ⤳ *If the businesswoman travels to the board meeting, it’ll be by plane*

To summarise our discussion thus far, the Extended Typology is built off a typology of proposed gesture types. Like gestures, there are three emoji types, distinguishable by their eliminability and alignment with the modified text, and semantically, by the inferences that they give rise to. The goal of the present study was to experimentally verify this extended typology. We conducted a set of four experiments aimed at investigating whether people draw the predicted types of inferences from the three types of emoji. Experiment 1 replicated a previous study by Pasternak and Tieu (2022), which tested the prediction that co-text emoji trigger cosuppositional inferences. Experiment 2 tested the claim that pro-text emoji trigger standard presuppositions, which project from embedded environments. Finally, Experiments 3 and 4 investigated post-text emoji, in particular testing whether they trigger supplemental inferences and whether this leads to degraded acceptability in the antecedents of conditionals.

### Experiment 1: co-text emoji

Pasternak and Tieu (2022) used an inferential judgement task (of the type already used to investigate linguistic inferences associated with gestures, as reported in Tieu et al., 2018, 2019), to investigate the projection of cosuppositional inferences of emoji. The authors tested five different emoji in six different linguistic environments, asking whether people would endorse the target cosuppositional inferences across the various linguistic environments. The main finding was that people indeed endorsed cosuppositional inferences, even in embedded environments such as negation and the scope of quantifiers, similar to what was reported for co-speech gestures by Tieu et al. (2018).

Experiment 1 replicated the emoji experiment reported by Pasternak and Tieu (2022). Given the goal of the overall study was to experimentally investigate the Extended Typology, it was, in our view, important to test the predictions for all three types of emoji, including co-text emoji. This also allowed for a more systematic comparison across the three emoji types, as we used similar methods and materials across the different experiments.

The key comparison in Experiment 1 involved the rates of endorsement of the target cosuppositional inferences when associated with target sentences compared to when they were associated with assertive control sentences. An example of an unembedded item and a negation item are provided in (24) and (25), respectively.

(24) a. *Target sentence*:
The student will 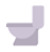 step out of the classroom 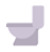
b. *Control sentence*:
The student will step out of the classroom to do this: 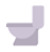
c. *Target inference*:

If the student were to step out of the classroom, it would be to use the toilet.

(25) a. *Target sentence*
The employee will not 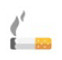 step out onto the balcony 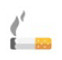
b. *Control sentence*:
The employee will not step out onto the balcony to do this: 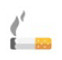
c. *Target inference*:
If the employee were to step out onto the balcony, it would be to smoke a cigarette.

While the unembedded target and control sentences in (24a) and (24b) might be expected to give rise to similar rates of endorsement of the target inference in (24c), in the case of negation, things are slightly different. While the target sentence in (25a) should trigger the cosuppositional inference in (25c), the assertive control sentence in (25b) explicitly denies the information expressed by the emoji. In the case of the negation condition, then, we should expect to see a difference between the endorsement rates of the target inference in the target condition compared to the control condition; that is, people should endorse the target inference in (25c) more for the target sentence in (25a) than for the control sentence in (25b).

### Participants

We tested 80 adults recruited through Prolific; 39 were randomly assigned to the target condition and 41 to the control condition. All participants self-reported as native speakers of English with normal or corrected-to-normal vision. Participants were paid at an average rate of £11/hr for the task, which took on average 11 m 25 s to complete.

### Procedure

**Figure 2.**
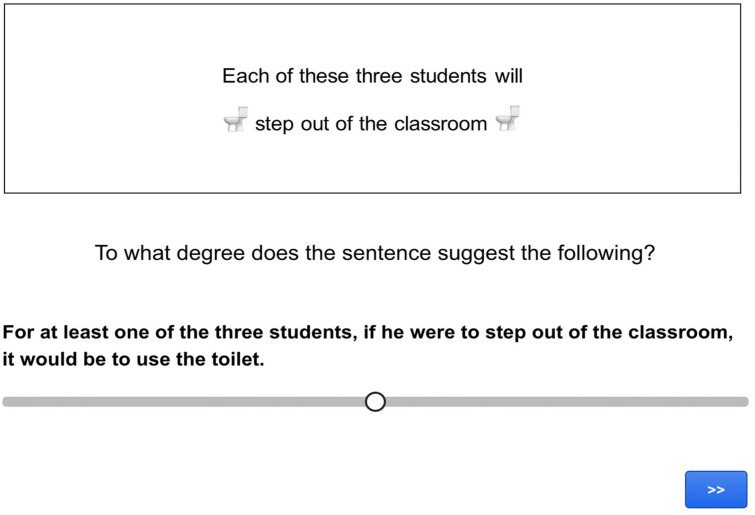
Screen capture of a co-text emoji target trial in Experiment 1.

**Figure 3.**
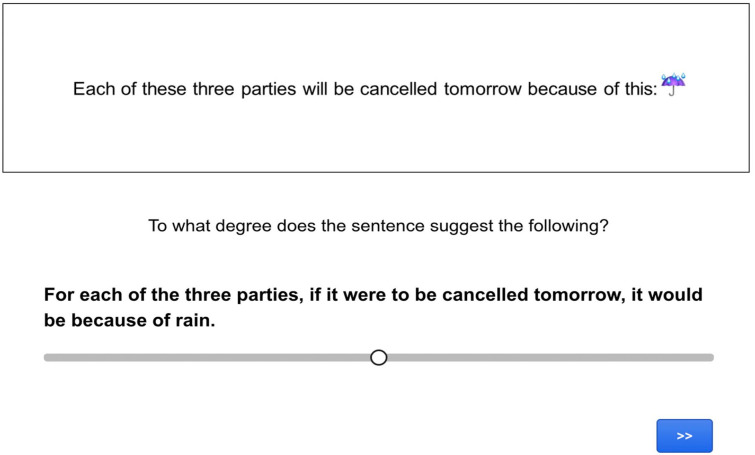
Screen capture of an assertive control trial in Experiment 1.

The procedure involved an inferential judgement task programmed using Gorilla Experiment Builder (www.gorilla.sc). In the task, participants had to read sentences containing an emoji and use a slider scale to judge how strongly the sentence led them to make a particular inference (see Figures 2 and 3 for screen captures of test and control trials).

### Materials

As in Pasternak and Tieu (2022), we tested five different emoji (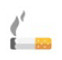, 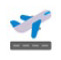, 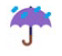, 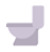, 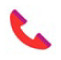) in six different linguistic environments: plain affirmative sentences (unembedded, with no logical operators) and five embedded environments corresponding to the scope of the modal “might”, the scope of negation, and the scope of the quantifiers “each”, “none”, and “exactly one”.

The unembedded items were as in (24), with the expectation of similar rates of endorsement of the target inferences for the target and control sentences. Example (26) illustrates the might condition. The control sentence in (26b) conveys that there is a possibility that the employee steps out onto the balcony to smoke a cigarette, even if it’s possible the employee might step out to do something else. The target sentence in (26a), on the other hand, conveys that if the employee steps out, it will be to smoke a cigarette—but not to do anything else. In the case of might, then, a difference could be observed in the endorsement of the target inferences in the target condition compared to the control condition.

(26) a. *Target sentence*:
 The employee might 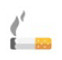 step out onto the balcony 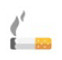
b. *Control sentence*:
 The employee might step out onto the balcony to do this: 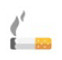
c. *Target inference*:
 If the employee were to step out onto the balcony, it would be to smoke a cigarette.

Consider next an example from the negation condition, repeated below in (27). While the target sentence in (27a) should give rise to the inference that stepping out onto the balcony would involve smoking a cigarette (27c), the assertive control explicitly asserts the opposite, namely that the employee will *not* step out onto the balcony to smoke a cigarette. In the case of negation, then, we should expect to see a significant difference between the target and the control sentences, in terms of endorsement of the target inference.

(27) a. *Target sentence*:
 The employee will not 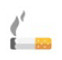 step out onto the balcony 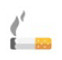
b. *Control sentence:*
 The employee will not step out onto the balcony to do this: 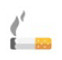
c. *Target inference:*
 If the employee were to step out onto the balcony, it would be to smoke a cigarette.

Consider next the quantifiers “each”, “none”, and “exactly one”. For each of these quantified environments, we tested both universal and existential inferences, as in the literature there are proponents of theories of universal projection (Heim, 1983; Schlenker, 2009) and of existential projection (Beaver, 2001), as well as reported experimental evidence for both (see Chemla, 2009 for evidence of universal projection under “none”, and Zehr et al., 2015, 2016 for more mixed data).

For the positive quantifier each, like in the unembedded case, similar inferences can be expected for the target (28a) and control (28b) sentences. In this case, both the universal (28c) and existential (28d) inferences are expected to be strongly endorsed.

(28) a. *Target sentence*:
 Each of these three employees will 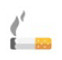 step out onto the balcony 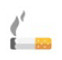
b. *Control sentence:*
 Each of these three employees will step out onto the balcony to do this: 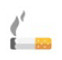
c. *Target universal inference:*
 For each of these three employees, if they were to step out onto the balcony, it would be to smoke a cigarette.
d. *Target existential inference:*
 For at least one of these three employees, if they were to step out onto the balcony, it would be to smoke a cigarette.

The none condition was expected to be similar to the negation condition in that the target and control sentences should give rise to different inferences. While the target sentence in (29a) conveys that none of the employees will step out onto the balcony, the assertive control in (29b) leaves open the possibility that some or all will step out onto the balcony—but asserts that it won’t be to smoke a cigarette. In the none environment, then, we expect differences between the targets and controls.

(29) a. *Target sentence*:
 None of these three employees will 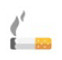 step out onto the balcony 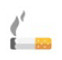
b. *Control sentence:*
 None of these three employees will step out onto the balcony to do this: 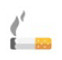
c. *Target universal inference:*
 For each of these three employees, if they were to step out onto the balcony, it would be to smoke a cigarette.
d. *Target existential inference:*
 For at least one of these three employees, if they were to step out onto the balcony, it would be to smoke a cigarette.

In the case of exactly one as well, differences may be expected between the targets and controls. The target sentence in (30a) requires that exactly one employee will step out, and that for all three employees, if they were to step out, it would be to smoke a cigarette. The control sentence in (30b), on the other hand, conveys that exactly one of the three employees will step out to smoke a cigarette—the other two might not step out at all or might step out to do something else. Here too, then, we might expect differences between the targets and controls.

(30) a. *Target sentence*:
 Exactly one of these three employees will 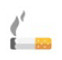 step out onto the balcony 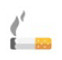
b. *Control sentence:*
 Exactly one of these three employees will step out onto the balcony to do this: 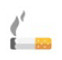
c. *Target universal inference:*
 For each of these three employees, if they were to step out onto the balcony, it would be to smoke a cigarette.
d. *Target existential inference:*
 For at least one of these three employees, if they were to step out onto the balcony, it would be to smoke a cigarette.

Half the participants were randomly assigned to the target condition, and half to the assertive control condition. Each participant saw a total of 45 items: five emoji in the unembedded environment, the same five emoji embedded under might, and the same five emoji embedded under negation. Each participant also saw the same five emoji embedded under three types of quantifiers: each, none, and exactly one. For the quantified cases, we tested for both universal and existential inferences; hence, there were twice as many quantified items (10 per quantifier).

### Results

**Figure 4.**
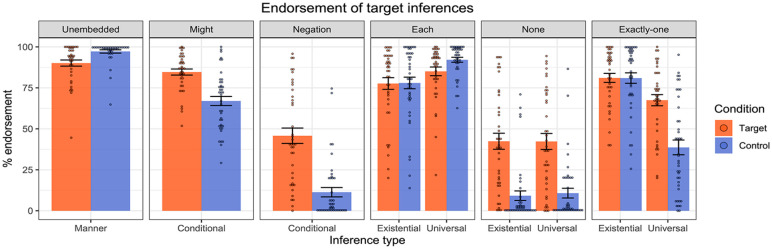
Results of Experiment 1—endorsement rates of target and control inferences across linguistic environments. Dots represent individual participants’ mean ratings.

**Figure 5.**
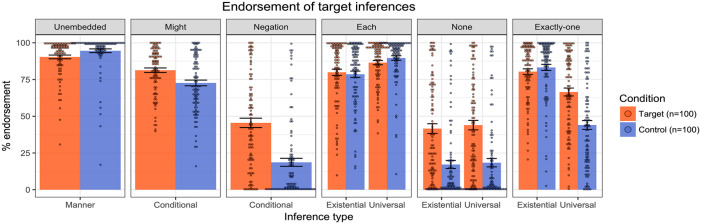
Results from Pasternak and Tieu’s (2022) emoji experiment.

Figure 4 presents the mean inferential judgements across the target and control conditions in each of the linguistic environments. Impressionistically, the results were remarkably similar to those reported in Pasternak and Tieu (2022) (see Figure 5), with generally strong endorsement of the target inferences in the four positive environments, unembedded, might, each, and exactly one, as might have been expected. For might and negation, as predicted, we observed stronger endorsement of the target inferences for the co-text targets compared to the assertive controls. Finally, for none and exactly one, we observed stronger endorsement of the universally projected target inferences for the target sentences compared to the assertive controls, consistent with the universal projection of the cosuppositional inferences. Importantly, the overall endorsement patterns largely mirror the earlier results from Pasternak and Tieu, with people endorsing and projecting cosuppositional inferences of co-text emoji.

For the planned analyses, we ran the same linear mixed-effect models as those reported in Pasternak and Tieu (2022). The idea was to test whether the target inferences were more strongly endorsed when associated with the target sentences containing the co-text emoji than when associated with the assertive/at-issue controls: this would indicate that the co-text emoji were behaving distinctly from mere at-issue modification. In the non-quantified environments (unembedded, might, negation), we fit mixed effect linear regression models to the inferential judgement data (using the lme4 package in R, R Core Team, 2016; Bates et al., 2015), with Condition (Target vs. Control) as a fixed effect, and random intercepts for participant and emoji. We then compared the maximal model in each case to the model without the fixed effect of Condition. In the unembedded condition, the model comparison revealed a significant effect of Condition, with stronger endorsement for controls than targets (χ^2^(1) = 11, *p* < .001). The model comparison in the might condition also revealed a significant effect of Condition, with stronger endorsement for targets than controls (χ^2^(1) = 24, *p* < .001). In the negation condition, we also observed a significant effect of Condition, with stronger endorsement of targets than controls (χ^2^(1) = 33, *p* < .001).

Moving to the quantified environments, we fit mixed effect linear regression models to the inferential judgement data, with Condition (Target vs. Control), Reading type (Existential vs. Universal) and their interaction as fixed effects, random by-participant slopes for Reading, and random intercepts for emoji. Starting with each, a model comparison did not reveal a significant interaction between Condition and Reading (χ^2^(1) = 2.3, *p* = .13), thus yielding no evidence that one reading was stronger in one condition than the other. We likewise observed no interaction in the case of none (χ^2^(1) = .34, *p* = .56). For exactly one, we observed a significant interaction between Condition and Reading (χ^2^(1) = 24, *p* < .001), indicating that one inference was stronger in one condition than the other. Follow-up models on the Existential data and the Universal data revealed no effect of Condition for the Existential data (χ^2^(1) = .0007, *p* = .98), but an effect for the Universal condition, (χ^2^(1) = 23, *p* < .001), with stronger endorsement of targets compared to controls.

To summarise, the findings perfectly replicate those of Pasternak and Tieu (2022). Co-text emoji trigger cosuppositional inferences which project from the scope of the modal “might”, negation, and the quantifiers “none”, “each”, and “exactly one”. Both sets of results suggest that co-text emoji can interact with logical structure in similar ways to gesture, moreover yielding patterns of behaviour that differ from mere at-issue modification such as “to do this: [emoji].”

## Experiment 2: pro-text emoji

The goal of our second experiment was to test whether pro-text emoji, which fully replace written words, can trigger standard presuppositions. To do so, we selected nine emoji that could signify a change of state: 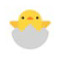, 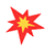, 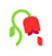, 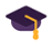, 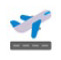, 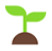, 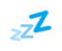, 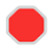, 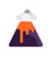. These nine emoji potentially trigger the presupposition that the pre-change state currently holds, as exemplified in the examples in (31)-(33), involving a pro-text emoji in a question, under negation, and under the negative quantifier “none”.

(31) Will the egg 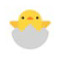
?
  ⤳ *The egg has not yet hatched*

(32) The plane will not 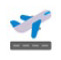  ⤳ *The plane is currently on the ground*

(33) None of these girls will 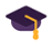  ⤳ *Each of these girls is currently a student*

### Participants

We recruited 60 adults (who had not completed Experiment 1) through Prolific. All self-reported as native speakers of English with normal or corrected-to-normal vision. Participants were paid at an average rate of £12.35/hr for the task, which took on average 12 m 54 s to complete.

### Procedure

**Figure 6.**
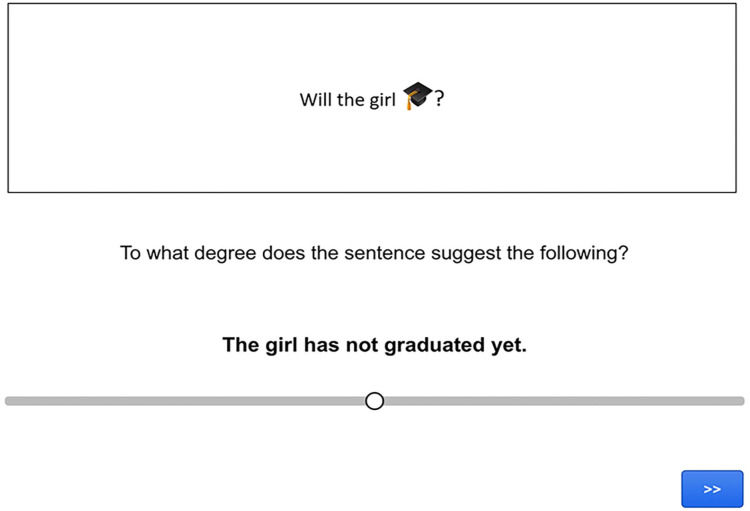
Screen capture of a pro-text emoji target trial in Experiment 2.

The task was again an inferential judgement task, in which participants had to read a sentence containing an emoji and use a slider scale to judge how strongly the sentence led them to make a particular inference (see Figure 6 for a screen capture of a trial).

### Materials

We tested the nine change-of-state emoji in five different linguistic environments. In addition to plain affirmative sentences, we included environments where standard presuppositions typically project: polar questions, the scope of negation, the modal “might”, and the scope of the quantifier “none”.

Each emoji sentence was paired with a target inference (the intended presupposition) and a baseline control inference, as in (34).

(34) a.  Will the egg 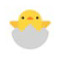
?
b. *Target inference*: The egg has not yet hatched.
c. *Control inference*: The egg has already hatched.

Inference type (Target vs. Control) was a within-subject factor. Each participant saw a total of 90 items, created by crossing the nine emoji with the five environments and the two types of inferences. The order of presentation of the items was completely randomised.

### Results

**Figure 7.**
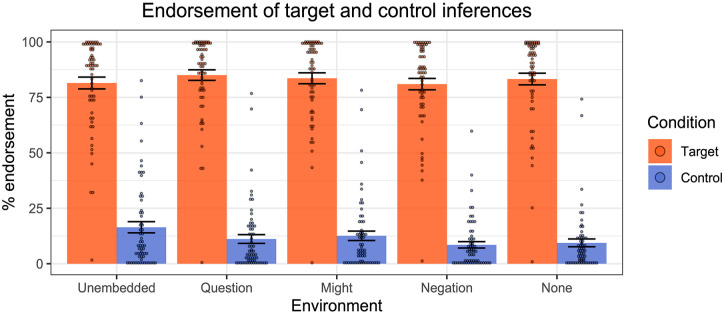
Results of Experiment 2—endorsement rates of target and control inferences across linguistic environments. Dots represent individual participants’ mean ratings.

The results across the various linguistic environments are displayed in Figure 7.

In general, participants endorsed the target inferences for the pro-text emoji more than the control inferences. We first fit a mixed-effect linear regression model to the inferential judgement data, with Condition (Target vs. Control) as a fixed effect and random by-participant slopes for Condition and random intercepts for emoji. A comparison of this model to one without the Condition factor revealed that Condition had a significant effect (χ^2^(1) = 123, *p* < .001), with stronger endorsement of targets than controls.

We then performed the same kinds of model comparisons for each of the linguistic environments individually. Across all environments we observed significantly higher endorsement for the Target condition compared to the Control condition (unembedded: χ^2^(1) = 89, *p* < .001; question: χ^2^(1) = 123, *p* < .001; might: χ^2^(1) = 113, *p* < .001; negation: χ^2^(1) = 132, *p* < .001; none: χ^2^(1) = 125, *p* < .001).

To summarise, people accessed presuppositions from pro-text emoji, and these projected from embedded environments, just as standard verbal presuppositions do. The data are therefore consistent with Pierini’s (2021) proposal about pro-text emoji: such emoji trigger presuppositions which project from the scope of negation, questions, the modal “might”, negation, and the quantifier “none”.

## Experiment 3: post-text emoji (inferences)

In the final two experiments, we turned our attention to post-text emoji, and in particular, the claim that post-text emoji, like post-speech gestures, trigger supplemental inferences, which are known to project from the antecedents of conditionals. In Experiment 3, we used an inferential judgement task to measure how strongly participants would endorse the target supplemental inferences, as in (35).

(35) If the businesswoman travels to the board meeting 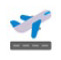, the company will reimburse her expenses.
⤳ *If the businesswoman travels to the board meeting, it’ll be by plane.*

To anticipate, we establish, based on the inferential judgement data, that people indeed draw supplemental inferences in such environments. In Experiment 4, we then investigate the further prediction that such supplemental inferences should result in degraded acceptability of post-text emoji in negative environments.

### Participants

We tested 60 adult participants recruited through Prolific who had not completed Experiment 1 or Experiment 2. All self-reported as native speakers of English with normal or corrected-to-normal vision. Participants were paid at an average rate of £10.90/hr for the study, which took on average 6 m 20 s to complete.

### Procedure

**Figure 8.**
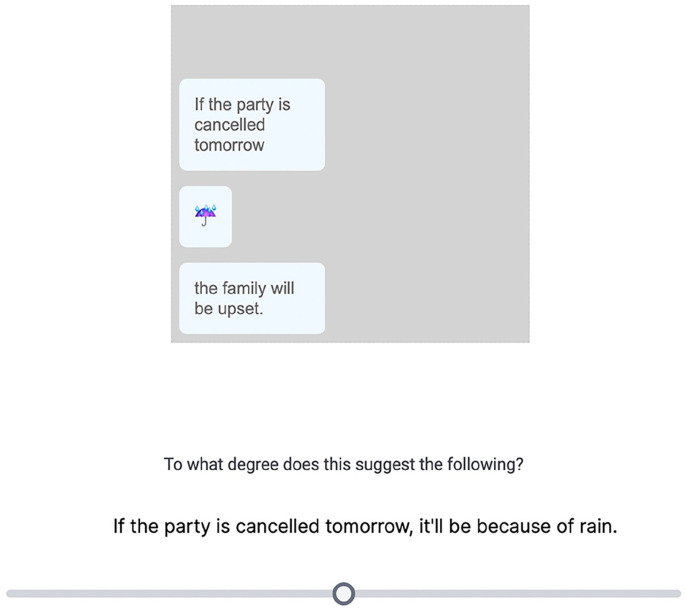
Screen capture of a post-text emoji target trial in Experiment 3. The text messages popped up one at a time, moving the sequence of messages upwards, mimicking the receipt of live texts.

The task was an inferential judgement task, as in Experiments 1 and 2. Participants read sentences containing post-text emoji and used a slider scale to judge how strongly the sentence led them to make a particular inference. In Gorilla Experiment Builder, we programmed the experiment so that the text messages would pop up one-by-one on the screen, mimicking the receipt of live texts. This helped to ensure that the post-text emoji unambiguously occupied its own time slot and would not be interpreted as a co-text emoji. In the example in Figure 8, for example, the text messages would pop up one at a time, pushing the sequence of messages upwards, as in a normal text messaging sequence.

### Materials

We tested five emoji in the antecedents of conditionals, as in (36)-(40), pairing each with a target inference (the intended supplemental inference) and a baseline control inference, to create 10 target items.

(36) a. *Target sentence*:
 If the employee steps out onto the balcony... 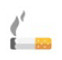, he will miss a phone call.
b. *Target inference:*
 If the employee steps out onto the balcony, it’ll be to smoke a cigarette.
c. *Control inference:*
 If the employee steps out onto the balcony, it’ll be to eat a snack.

(37) a. *Target sentence:*
 If the party is cancelled tomorrow ... 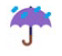, the family will be very disappointed.
b. *Target inference:*
 If the party is cancelled tomorrow, it’ll be because of rain.
c. *Control inference:*
 If the party is cancelled tomorrow, it’ll be because of snow.

(38) a. *Target sentence:*
 If the professor is interrupted during her lecture ... 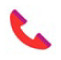, she will end the lecture early.
b. *Target inference:*
 If the professor is interrupted during her lecture, it’ll be because of a ringing phone.
c. *Control inference:*
 If the professor is interrupted during her lecture, it’ll be because of a fire alarm.

(39) a. *Target sentence:*
 If the businesswoman travels to the board meeting ...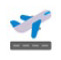, the company will reimburse her expenses.
b. *Target inference:*
 If the businesswoman travels to the board meeting, it’ll be by plane.
c. *Control inference:*
 If the businesswoman travels to the board meeting, it’ll be by train.

(40) a. *Target sentence:*
 If the student steps out of the classroom ...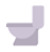, the teacher will pause the class.
b. *Target inference:*
 If the student steps out of the classroom, it’ll be to use the toilet.
c. *Control inference:*
 If the student steps out of the classroom, it’ll be to use the phone.

In addition to the 10 target items, we included 10 filler items, which corresponded to the five pro-text emoji from Experiment 2 (each with their respective target and control inferences), for a total of 20 items.

### Results

**Figure 9.**
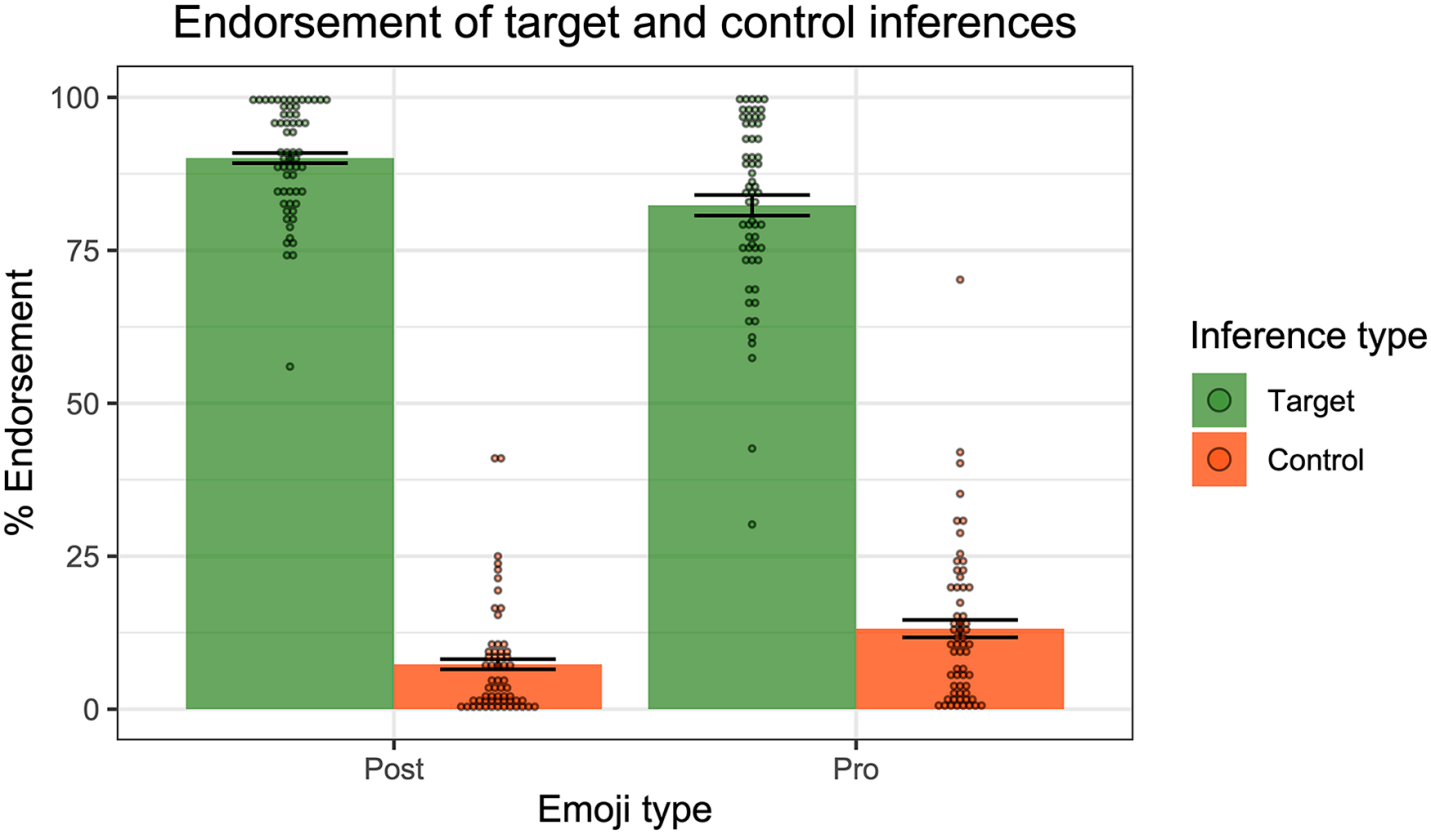
Results of Experiment 3—endorsement rates of target and control inferences for post-text emoji targets and pro-text emoji fillers. Dots represent individual participants’ mean ratings.

Figure 9 displays the mean rates of endorsement of the target and control inferences for the post-text targets and pro-text fillers. In general, people agreed more with the target inferences than with the control inferences, for both the post-text targets and pro-text fillers. We fit a mixed effects linear regression model to the data from the post-text target condition, with Condition (Target vs. Control) as a fixed effect, random by-participant slopes for Condition, and random intercepts for emoji. We then performed a model comparison, which revealed a significant effect of Condition, with higher endorsement of target inferences than control inferences (χ^2^(1) = 212, *p* < .001).

To summarise the results of Experiment 3, we found that people strongly endorsed supplemental inferences from post-text emoji in the antecedents of conditionals—consistent with the predictions of the Extended Typology.

## Experiment 4: post-text emoji (acceptability)

Next, we investigated the predictions for the acceptability of post-text emoji. If post-text emoji do indeed contribute supplements, as seems to be supported by Experiment 3, then we should also predict that they should be degraded in negative environments because supplements are generally unacceptable in negative environments (see Potts, 2005). We used an acceptability judgement task to compare the acceptability of post-text emoji in positive and negative sentences like the pair in (41).

(41) a. The party will be cancelled tomorrow ...
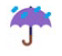
b. #The party will **not** be cancelled tomorrow ...
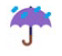

A sentence like (41a) should be relatively natural, conveying that the party will be cancelled because of rain. By contrast, the negative counterpart (41b) is predicted to be degraded in acceptability.

Note that if participants were to find the positive cases more acceptable than the negative cases, we might conclude that post-text emoji indeed contribute supplements, hence the lower acceptability ratings in negative environments. However, such an effect of polarity could also simply be due to the overall lower endorsement of negative sentences. That is, people might simply like negative sentences less than positive ones.^7^

To control for this, we included co-text controls. Co-text emoji provide an ideal control: the sentences with the co-text emoji would be minimally different from the post-text target sentences (with the only difference being the alignment/time slot of the emoji), but crucially for co-text emoji, there is no expectation of a (degradation) effect of negation. In addition to the positive and negative post-text targets, then, we also included positive and negative control sentences with co-text emoji, as in (42).

(42) a. The party will 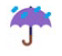 be cancelled tomorrow 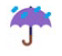
b. The party will **not**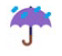 be cancelled tomorrow 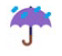

An interaction between emoji type (post- vs. co-text) and polarity (positive vs. negative) would importantly tell us that an observed degradation effect was not merely due to the presence of negation. In other words, one should expect to see a greater degradation effect of negation for post-text emoji (due to the hypothesised presence of supplements) than for co-text emoji.

### Participants

61 adults who had not completed Experiments 1, 2, or 3, were recruited through Prolific. All participants self-reported as native speakers of English with normal or corrected-to-normal vision. They were paid at an average rate of £13.53/hour for the study, which took on average 11 m 9 s to complete.

### Procedure

**Figure 10.**
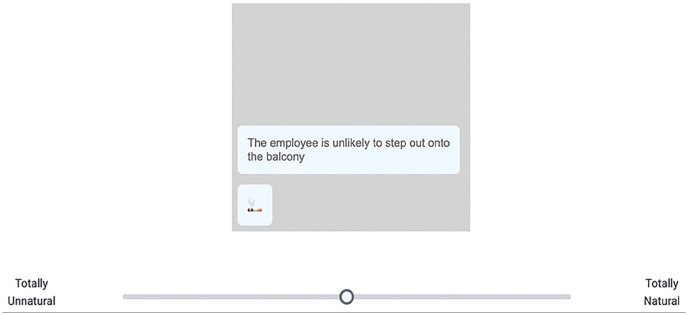
Screen capture of a post-text emoji target trial in Experiment 4. The text messages popped up one at a time, mimicking the receipt of live texts.

The task was a linear acceptability judgement task. Participants had to read sequences of text messages containing emoji (similar to those in Experiment 3) and use a slider scale to judge how natural the utterances were (see Figure 10). The scale was not numerically labelled; the endpoints were labelled “Totally Unnatural” and “Totally Natural”. In the instructions to participants, we included an example of a clearly acceptable sentence with an emoji, an example of an incongruent use of an emoji, and an example that participants were told might be harder to judge (the instructions that were presented to participants are part of the shared materials for this study, see “Data accessibility statement”).

As in Experiment 3, to ensure that the post-text emoji unambiguously occupied their own time slot and were thus distinguishable from co-text emoji, the text messages were programmed to pop up dynamically to mimic the receipt of live texts.

### Materials

Each participant saw a total of 60 items: 30 targets (5 post-text emoji × 3 environments × 2 polarities), and 30 controls (5 co-text emoji × 3 environments × 2 polarities), presented in a fully randomised order.

### Results

**Figure 11.**
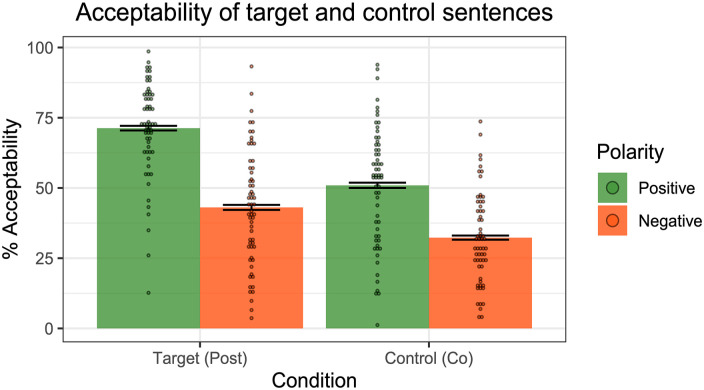
Results of Experiment 4—acceptability ratings of positive and negative post-text emoji targets and co-text emoji controls. Dots represent individual participants’ mean ratings.

Figure 11 displays the mean acceptability ratings for the target and control sentences across polarities.

Participants generally gave higher ratings to positive sentences compared to negative sentences. We fit a mixed effect linear regression model to the data, with Condition (Target vs. Control), Polarity (Positive vs. Negative) and their interaction as fixed effects, random by-participant slopes for Condition, and random intercepts for Emoji type and Environment. A model comparison revealed a significant interaction between Condition and Polarity (χ^2^(1) = 50, *p* < .001), with a greater difference between polarities for post-text emoji than co-text emoji. That is, adding negation led to a larger degradation in acceptability for post-text emoji than for co-text emoji. This interaction importantly tells us that the degradation effect goes beyond a general dispreference for negative sentences.

**Figure 12.**
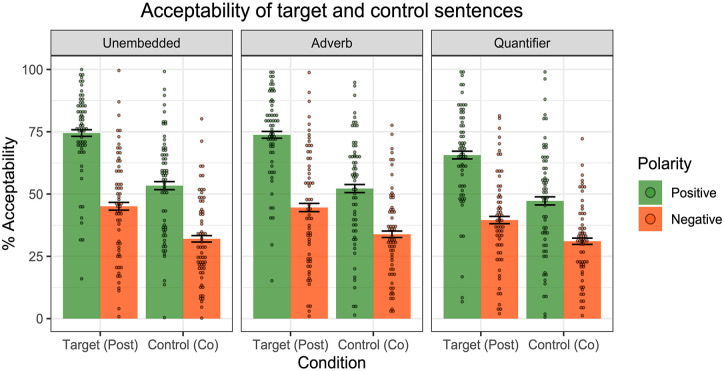
Results of Experiment 4—acceptability ratings across the three linguistic environments. Dots represent individual participants’ mean ratings.

Figure 12 displays the acceptability ratings for the post-text targets and co-text controls across the three linguistic environments. A significant interaction between Condition (Target vs. Control) and Polarity (Positive vs. Negative) was observed in each of the environments (Unembedded: χ^2^(1) = 15, *p* < .001; Adverb: χ^2^(1) = 29, *p* < .001; Quantifier: χ^2^(1) = 21, *p* < .001). In short, for all three examined linguistic environments, negation had a greater degradation effect on post-text emoji compared to the co-text emoji.

To summarise Experiment 4, participants indeed judged post-text emoji to be more acceptable in positive sentences compared to negative sentences. The difference across polarities was found to be more pronounced for post-text emoji compared to our co-text emoji controls. The observed interaction between emoji type and polarity suggests that the degradation effect goes beyond a general dispreference for negative sentences. One line of explanation, consistent with Pierini’s proposal for post-text emoji, is that post-text emoji trigger supplements, which are reported to be worse in negative linguistic environments.

## General discussion

In this paper, we presented a set of four experiments designed to put Pierini’s (2021) Extended Typology to the test. Experiment 1 tested the hypothesis that co-text emoji trigger cosuppositional inferences that project from embedded environments like standard verbal presuppositions do. The results replicated those reported in Pasternak and Tieu (2022), with participants endorsing and projecting cosuppositional inferences. Note that participants responded to the co-text emoji in entirely the same way that people have been reported to respond to co-speech gestures (Tieu et al., 2018) and co-speech sound effects (Pasternak & Tieu, 2022). Importantly, in the latter two cases, the relevant stimuli could and did occur simultaneously with the relevant modified portion of the speech. The parallel results across the three modalities reassure us that people did indeed interpret the bracketed emoji as co-text emoji, in parallel with co-speech gestures and co-speech sound effects.

Experiment 2 tested the hypothesis that pro-text emoji, which fully replace words, can trigger presuppositions. Participants indeed endorsed the presuppositional inferences of the pro-text emoji, in both plain positive sentences and more complex sentences from which presuppositions typically project. Finally, Experiments 3 and 4 focused on post-text emoji. Experiment 3 tested the hypothesis that post-text emoji trigger supplements, and Experiment 4 tested the further consequence that post-text emoji should be degraded in negative linguistic environments. Participants indeed endorsed the supplemental inferences in Experiment 3, and in Experiment 4, judged negative sentences containing post-text emoji to be worse than positive sentences containing post-text emoji.

One area worth revisiting in future work pertains to the co-text emoji sentences that we used as a control in Experiment 4. Although these were included as an “acceptable control” (since there is no theoretical reason to expect any degradation effect of polarity for co-text emoji), the acceptability judgements for the co-text emoji were generally not very high (in positive or negative environments). We suspect the lower ratings were because participants found the emoji bracketing hard to judge out of context. Experiment 1, which also involved bracketed emoji, yielded sensible results, because an inferential judgement task essentially requires that one imagine situations in which the text messages could be produced. In the acceptability judgement task in Experiment 4, on the other hand, people were simply presented with the bracketed emoji out of the blue, and asked to judge their naturalness. It is possible that one might obtain higher acceptability judgements by presenting the sentences in context, perhaps using the target cosuppositional inferences that we tested in Experiment 1. For example, the co-text emoji in (43) might be judged as more natural if presented with a preceding context, compared to on its own.

(43) *Context: If the employee steps out onto the balcony, it’ll be to smoke a cigarette*.
The employee will 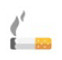 step out onto the balcony 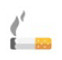

We leave for future research the question of the general acceptability of emoji (and co-text emoji in particular).^8^ What is relevant for the purposes of the current study is the observed difference in the effect of polarity across the two kinds of emoji.

The results from the four experiments presented here lend support to the Extended Typology. Strikingly, the proposed typology builds on Schlenker’s (2018b) typology of gestures, and thus the present findings provide further evidence of the parallels between gestures and emoji already noted in previous work (for example, Gawne & McCulloch, 2019; Miyake, 2007; Schandorf, 2013). Finally, the present study contributes to a growing line of research that applies formal linguistic methods and analyses to apparently non-linguistic objects such as emoji (see Patel-Grosz et al., 2023 for an overview of this “super-linguistic” approach). Such research can provide non-trivial insights into the core properties shared across surprisingly diverse modalities.

## Notes

1. Much formal semantic work on gestures (and on co-speech gestures in particular) has focused on the precise meaning contributions of gestures and the ways in which they interact with the logical structure of the sentences they modify (Anvari, 2017; Davidson, 2015; Ebert & Ebert, 2014; Esipova, 2018, 2019; Hunter, 2019; Lascarides & Stone, 2009; Schlenker, 2018a, 2018b; Tieu et al., 2017, 2018; Zlogar & Davidson, 2018).

2. The images in examples (11) and (12) are reproduced from Schlenker (2019) with permission.

3. The actual use of this bracketing structure for emoji seems to be somewhat limited in naturally occurring samples of emoji. The *sparkle* emoji  seems to be a notable exception to this, with recent work arguing that the sparkle emoji can bracket words or phrases to indicate focus (Kaiser, 2021; see also Broni, 2021 for relevant discussion). Another potentially related example we have observed is the use of the musical note emoji  to bracket song lyrics or a portion of text that is meant to be sung. For the purposes of our experiment (which did not involve the sparkle emoji), the bracketed emoji provided a sufficiently close equivalent to co-speech gestures, by transparently marking the part of the text that was meant to be modified by the emoji.

4. (13a) may look more natural than (13b). However, one problem with the version in (13a) is that it becomes difficult (if not impossible) to distinguish it from a post-text version, where the emoji would necessarily have to follow the written sentence. One of our pilot experiments included sentence-final emoji such as (13a), compared to a “post-text” version in which the emoji appeared in a subsequent text message; participants treated the two kinds of sentences identically, suggesting that (13a) more likely leads to a “post-text” treatment of the emoji, rather than a “co-text” one.

5. Cohn et al. (2019, Exp. 2) asked participants to produce textual utterances in which at least one emoji was substituted for words. They report that emoji were substituted more for nouns and adjectives than verbs, and moreover that such emoji substitutions typically conveyed nonredundant information. For the purposes of testing presuppositional meanings, our Experiment 2 will make use of emoji that can convey actions (thus replacing the verbs in the sentences).

6. The present study focuses on emoji that denote objects and activities; we set aside the topic of face emoji, including sentence-final face emoji, which reportedly can be used to convey irony (see Weissman & Tanner, 2018 for psycholinguistic evidence that sentence-final emoji can trigger the same kinds of neural responses as word-generated irony). See also Grosz et al. (2023) for an analysis of the semantics of face emoji.

7. Psycholinguistic studies have shown that out of the blue, negative sentences are harder to process compared to their positive counterparts (Wason, 1961; for an overview, see Tian & Breheny, 2019). See also Davidson (2023) for relevant evidence and discussion pertaining to the relative unacceptability of iconic depictions (which can include co-speech gestures) under negation.

8. To our knowledge, no studies have investigated the relative acceptability of co-, pro, and post-text emoji, though previous studies have made use of acceptability judgement tasks to investigate the possible meanings and functions of emoji (see, e.g., Bjertnes, 2022; Hamdan, 2022; Paggio & Tse, 2022). Paggio and Tse (2022) present evidence that emoji that are used as word replacements (pro-text emoji, in our terminology) may be hard for readers to process, particularly when their meaning is not immediately clear. But there are no studies yet that directly compare the acceptability of pro-text and co-text emoji; the relative acceptability of the different types of emoji thus remains an area ripe for further research.
